# Expression and Significance of MyD88 in Patients With Gastric Cardia Cancer in a High-Incidence Area of China

**DOI:** 10.3389/fonc.2020.00559

**Published:** 2020-05-14

**Authors:** Jingyao Chen, Di Xia, Muming Xu, Ruibing Su, Wenting Lin, Dan Guo, Guangcan Chen, Shuhui Liu

**Affiliations:** ^1^Department of Pathology, Shantou University Medical College, Shantou, China; ^2^Department of Abdominal Surgery, The Tumor Hospital of Shantou University Medical College, Shantou, China; ^3^Department of Gastrointestinal Surgery, The First Affiliated Hospital of Shantou University Medical College, Shantou, China

**Keywords:** MyD88, *Helicobacter pylori*, gastric cardia cancer, cancer and inflammation, prognosis

## Abstract

**Background:** Gastric cardia cancer (GCC) arises in the area of the stomach adjoining the esophageal–gastric junction and has unique risk factors. It was suggested that the involvement of *Helicobacter pylori* is associated with GCC from high-risk population. Myeloid differentiation factor 88 (MyD88) is a crucial adaptor molecule in Toll-like signaling pathway recognizing *H. pylori*. Its role in GCC has not been elucidated yet. In this study, our purpose is to investigate the expression and significance of MyD88 in GCC tissue.

**Methods:** Expression of MyD88 and nuclear factor κB (NF-κB) p105/p50 and infection of *H. pylori* were detected by immunohistochemistry in gastric cardia tissue. The correlation of MyD88 expression to NF-κB p105/p50 expression, *H. pylori* infection, and clinicopathologic characteristics in gastric cardia tissue was analyzed. The involvement of MyD88 in patient prognosis was also analyzed.

**Results:** Our data showed that the expression of MyD88 elevated from normal mucosa to inflammation (*p* = 0.071). The expression of MyD88 was enhanced in GCC tissues by contrast to non-malignant cardia mucosa (*p* = 0.025). What's more, overexpression of MyD88 was detected in intestinal-type adenocarcinoma with inflammation. Patients with high MyD88 staining revealed a better differentiation (*p* = 0.02). MyD88 also positively correlated with NF-κB p105/p50 expression (*p* = 0.012) in cancer tissue. Expression of MyD88 was increased but not significantly in biopsies with *H. pylori* infection compared with non-infected biopsies. Multivariate analyses revealed lymph node metastasis but not MyD88 expression was an independent predictor for patient survival.

**Conclusion:** These findings provide pathological evidence that upregulating MyD88 and inducing inflammation might be involved in gastric cardia carcinogenesis in high-risk population. MyD88 plays a role in gastric cardia carcinogenesis with NF-κB pathway activation. Higher MyD88 expression is not a major prognostic determinant in GCC, but it may relate to the tumor cell differentiation.

Gastric cancer is a significant global health problem. It is one of the five most common malignancies and ranks after lung, breast, colorectal, and prostate cancer in 2012 (1). Geographically, 43% of total global cases occur in China (1). Gastric cancer is generally divided into two topographical categories: gastric cardia cancer (GCC) arising in the upper part of the stomach, where it connects to the esophageal, and non-GCC (NGCC) arising from rest part of the stomach. Gastric cardia cancer has unique epidemiology and risk factors different from NGCC. The incidence of GCC has been stable or increased, and the NGCC incidence decreased since the mid-1970s (1).

In China, the incidence of GCC differs on the basis of geographical situation and populations. Gastric cardia cancer has epidemiologic features of population and familial aggregation. The regions in China with high incidence rate of esophageal cancer also have high incidence of GCC, such as Linzhou (2) and Chaoshan area (3). Different from GCC, the incidence of NGCC is low in these areas. Risk factors of GCC are unclear and controversial. Studies of Caucasian populations suggested risk factors for GCC are similar to those for esophageal adenocarcinoma, including obesity, gastroesophageal reflux disease, and Barrett esophagus (1). *Helicobacter pylori* with positive test associated with NGCC is suggested inversely associated with GCC in Western countries. However, in high-risk settings, a positive association between *H. pylori* infection and gastric cancer was observed both for cardia and non-cardia cancers (4). Reports showed that the influence of gender, socioeconomic status, presence of intestinal metaplasia, and past alcohol intake also differ in GCC and NGCC (1). Considering the differences, more and more researches are addressing GCC and NGCC as separate diseases.

The Chaoshan GCC high-incidence area of east Guangdong province is the only coastal high-incidence area in China. From 1995 to 2004, previous epidemiological data revealed that the incidence of GCC was unusually high (34.81/100,000) on Nan'ao Island in the Chaoshan area (3). Our previous researches found that *H. pylori* infection accompanied with chronic inflammation may result in the carcinogenesis of GCC in Chaoshan region (5, 6).

Toll-like receptors (TLRs) may acquire oncogenic potential by initiating inflammatory pathways, which are essential for *H. pylori* recognition (5–7). The TLRs transmit signals through adaptor proteins. The first adaptor molecule of TLRs to be discovered is myeloid differentiation factor 88 (MyD88) (8). MyD88 is essential in regulating innate immune signals from members of the TLR and interleukin families. Toll-like receptors and interleukin 1 receptors can recognize microbes or endogenous ligands and then recruit MyD88, which can induce nuclear factor κB (NF-κB) activation (8–12). Previous study suggested that abnormal expression of MyD88 was closely associated with the development of tumor and resistance of drugs. In stomach, lung, liver, ovary cancer tissues, the expression of MyD88 was enhanced (8). However, the research data are contradictory. The effects of MyD88 in the development and progression of cancers are controversial (13, 14). MyD88-deficient mice models have shown MyD88 may either promote (10, 15–17) or suppress (18–20) tumor development. In colon cancer models, MyD88 showed contradictory roles even in the same cancer (21, 22).

Our previous study suggested that TLR4 plays a role in carcinogenesis of Chaoshan GCC (7). However, the expression of MyD88 in GCC and its effects on GCC development remain unknown. In the present study, we investigate the expression of MyD88 in gastric cardia tissue of different lesions from Chaoshan high-risk area and evaluate its correlation with *H. pylori* infection and NF-κB pathway activation.

## Materials and Methods

### Study Patients

One hundred two gastric cardia carcinoma samples and 95 non-malignant gastric cardia mucosa were obtained from the Tumor Hospital and the First Affiliated Hospital of Shantou University Medical College in Chaoshan area. The inclusion criterion for GCC is the center of cancer within 2 cm below the gastroesophageal junction defined by the World Health Organization (23). Follow-up survey was conducted for survival status of 71 patients by mobile phone or personal interview. Table 3 shows the clinicopathological features of the GCC patients. The median age was 62 years with range 40–78 years. Mean tumor diameter was 6 cm (range, 3–15 cm). In this study, we obtained all patients' informed consent and approval from the ethical review committees of the Medical College of Shantou University.

### Immunohistochemistry

Formalin-fixed paraffin-embedded samples were sectioned at 4 μm and deparaffinized with xylene and rehydrated with graded ethanol, and then 3% hydrogen peroxide was used for preincubating for 10 min. Antigen retrieval was performed by heating for 20 min in microwave oven. Then, the sections were incubated with 10% normal goat serum to block/eliminate non-specific staining. Next, the tissues were incubated overnight at 4°C with the following antibodies: anti-MyD88 rabbit monoclonal antibody (ab133739; Abcam; Cambridge, MA, United States), anti–NF-κB p105/p50 rabbit monoclonal antibody (ab32360; Abcam; Cambridge, MA, United States), or anti-*H. pylori* rabbit polyclonal antibody (RAB-0064; Fuzhou Maixin Biotechnology; Fuzhou, Fujian Province, China). The tissues were incubated with the secondary antibody conjugated with horseradish peroxidase at 37°C for 30 min. Sections were counterstained with hematoxylin and mounted with glycerol gelatin. We used Olympus BX43 microscope (Olympus, Japan) and Olympus DP21 image management system (Olympus, Japan) to capture images.

Immunohistochemistry (IHC) staining score was evaluated by two experienced researchers in a blinded manner. The expression of MyD88 and NF-κB p105/p50 was rated (0–3) semiquantitatively according to the signal intensity (0 = no immunostaining, 1 = weak positive staining, 2 = moderate positive staining, 3 = strong positive staining) (24). We found intensity in different areas of the specimen was different. We observed the whole specimen and counted all positive and negative cells. Most sections with total number of cells varied from 5,000 to 8,000. The value was calculated by multiplying the scores of staining intensity by the proportion of positive cells (0–100%).

All values were added to generate a final score ranging from 0 to 300 (25). *Helicobacter pylori* IHC–positive test showed *H. pylori* are brownish yellow, thick, and rod-like, and some of them are clumps (26).

### Chronic Inflammation Grading

According to the updated Sydney System, chronic inflammation was measured by the presence of polymorphonuclear leukocytes alongside the mononuclear inflammatory infiltration. The normal gastric mucosa has fewer than 5 inflammatory cells in the lamina propria. Mild inflammation shows 5–30 inflammatory cells in the lamina propria per high-power field or the foveolae. More than 30 inflammatory cells per high-power field infiltrating mucosal layer was considered severe inflammation (27).

### Statistical Analysis

Independent non-parametric *t*-tests for trend were used to evaluate increased or decreased MyD88 expression among groups. Spearman correlation was used to determine the correlation between MyD88 and NF-κB p105/p50. The risk factors for overall survival were tested by a Cox proportional hazards model with a stepwise forward procedure. All statistical analyses were performed by using SPSS v19 (SPSS, Chicago, IL, USA). *P* < 0.05 was considered statistically significant.

## Results

### MyD88 Expression in Gastric Cardia Tissue

**Figure 1:**
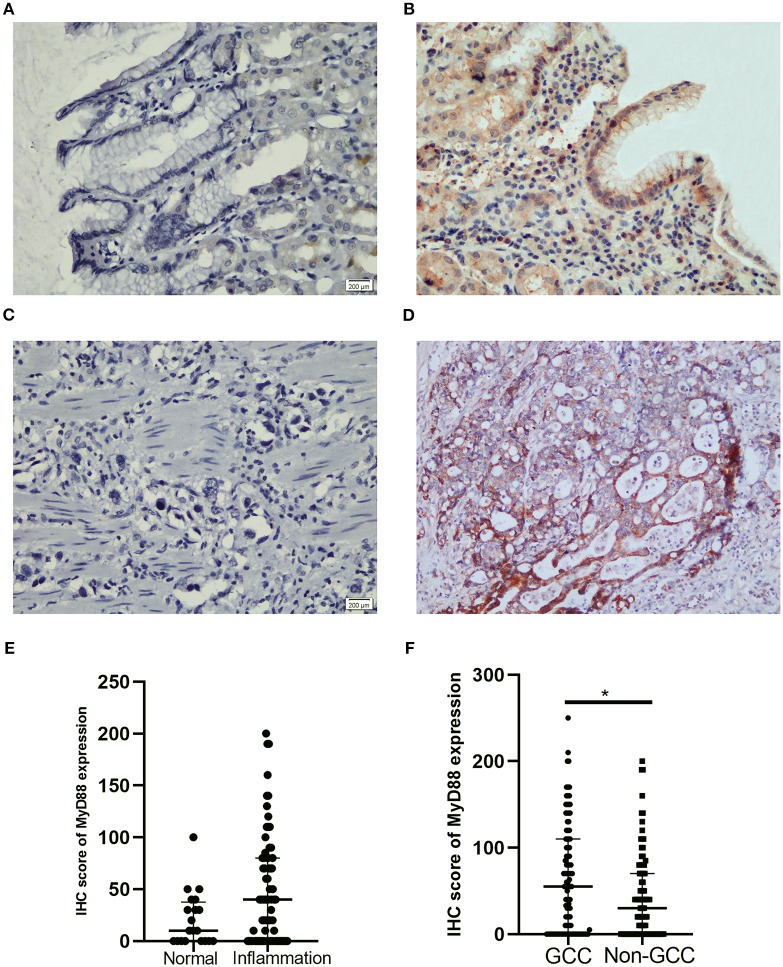
Representative IHC staining for MyD88 in gastric cardia mucosae and gastric cardia cancer tissue. **(A)** No immunostaining in normal mucosae, **(B)** moderate positive staining in mucosae with inflammation, **(C)** negative MyD88 staining in poorly differentiated tumor, and **(D)** strong positive staining in intestinal-type adenocarcinoma. **(E)** Boxplot shows MyD88 expression was higher in the inflammation cases than normal tissues. **(F)** Boxplot shows MyD88 expression was significantly higher in the GCC cases compared to the non-malignant cases (*p < 0.05).

To detect the expression of MyD88 in nonmalignant tissue and GCC tissue, we performed MyD88 immunohistochemical staining in this retrospective cohort study. In Table 1 and Figure 1, MyD88 expression in the different gastric cardia lesions is shown. Immunostaining of MyD88 protein was mainly found in the cytoplasm, which was consistent with published result (28). Among the non-malignant gastric cardia tissues, MyD88 expression was higher in inflamed epithelia than that in normal gastric cardia mucosa (Figures 1A,B), and the *p*-value was close to significant level, but did not differ between mild and severe inflammation. In 102 GCC cases, MyD88 expression was detectable in most of the cases 72/102 (70.59%). MyD88 expression was significantly higher in tumor tissue than that in non-malignant gastric cardia tissues (*p* = 0.025) (Table 1). Moreover, the stronger MyD88 staining was found in intestinal-type adenocarcinoma with severe inflammation than in diffuse-type cancer (Figures 1C,D).

**Table 1:** Immunohistochemical evaluation of MyD88 expression in different gastric cardia tissue.

**Tissue feature**		**Case**	**MyD88 expression, percentage of positive cells (%)**	**Myd88 expression, median (IQR)**	***P*-value**
Tumor or non	GCC	102	38.93	55 (0, 110)	*p* = 0.025^*^
	Non-GCC	95	26.45	30 (0, 70)	
*H. pylori* infection	Negative	63	24.1	20 (0, 60)	*p* = 0.228
	Positive	32	31.01	40 (0, 80)	
Inflammation	Normal	20	17.75	10 (0, 37.5)	*p* = 0.071
	Mild/severe	75	28.77	40 (0, 80)	

* *p < 0.05*.

### Correlation Between MyD88 Expression and *H. pylori* Infection

**Figure 2:**
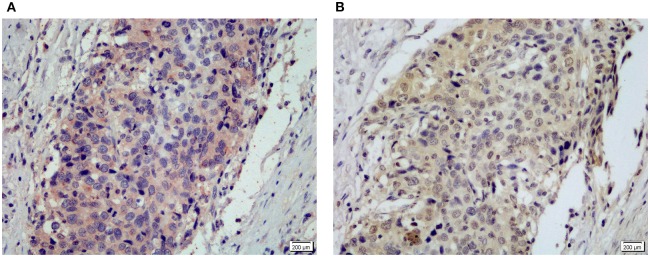
Representative IHC staining for MyD88 **(A)** and NF-κB p50/105 **(B)** in the same gastric cardia cancer tissue.

MyD88 may play a role in gastric immunologic response to *H. pylori* (29, 30). We hypothesized that MyD88 expression correlates with *H. pylori* infection. At the beginning we tried to compare the MyD88 expression correlates with *H. pylori* infection in GCC tissue. However we found that most of the tumor tissue had necrosis and very few samples could found *H. pylori* by IHC. So we just used the non-GCC tissue to analyze *H. pylori* infection. We think that the results from non-malignant gastric cardia tissues can reflect the relationship between MyD88 expression and *H. pylori* infection. Thus, we use immunohistochemical staining to detect *H. pylori* infection in the non-malignant gastric cardia tissues. *Helicobacter pylori* was seen in the mucosa and gland epithelium tissues (Figure 2A). Expression of MyD88 was increased in biopsies with *H. pylori* infection compared with non-infected biopsies; however, the difference was not significant (Table 1, Figure 2B).

### Correlation Between MyD88 Expression and NF-κB in GCC

**Figure 3:**
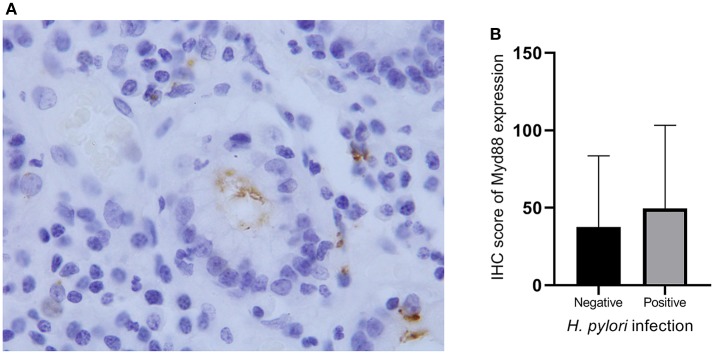
*Helicobacter pylori* infection and MyD88 expression. **(A)** *Helicobacter pylori* infection was identified by IHC in gastric cardia glands. **(B)** Boxplot shows that MyD88 expression was higher in the severe *H. pylori* infection cases than the cases without *H. pylori* infection but not significantly.

MyD88 plays an important role in tumor immunity by regulating NF-κB–mediated functions (8, 31). We used an antibody that can recognize both p105 and p50 proteins to quantify NF-κB p105/p50 protein in the same cohort of samples. Immunohistochemical staining detected NF-κB p105/p50 in all non-malignant and malignant samples. Expression of NF-κB p105/p50 was higher in GCC (*n* = 104) than in non-malignant tissues (*n* = 94) (*p* = 0.000). Moreover, increased NF-κB p105/p50 staining in gastric cardia tissues was positively associated with overexpression of MyD88 expression (*p* = 0.012) (Table 2). The strongest immunostaining of NF-κB p105/p50 and MyD88 coexisted in tumors (Figure 3).

**Table 2:** Correlation of MyD88 and NF-κB p50/105 expression in gastric cardia cancer tissue.

		**NF-κB p50/105 (*n* = 102)**
MyD88 (*n* = 102)	Correlation	0.248
	*P*-value	0.012^*^

* *p < 0.05*.

### Clinical Significance of MyD88 Expression in Gastric Cardia Cancer Patients

We then analyze the relationship between MyD88 expression and clinicopathologic features of GCC patients including gender, size of the tumor, lymph node metastasis, histological grade, depth of tumor invasion, and TNM stage (Table 3).

**Table 3:** The associations of MyD88 expression with clinicopathologic characteristics concerning 71 of the 102 GCC patients.

**Features**	**MyD88 expression, percentage of positive cells (%)**	**MyD88 expression, median (IQR)**	***P*-value**
**Gender**			0.475
Male (*n* = 63)	43.26	70 (10, 120)	
Female (*n* = 8)	41.25	35 (2.5, 87.5)	
**Age**			0.934
≤ 62 (*n* = 33)	42	63 (0, 150)	
>62 (*n* = 37)	43.51	70 (20, 100)	
**Size**			0.803
<6 cm (*n* = 36)	42.94	66.5 (10, 135)	
≥6 cm (*n* = 35)	43.14	60 (0, 100)	
**Tumor differentiation**			0.02^*^
Well/moderately (*n* = 39)	53.23	70 (40, 130)	
Poorly (*n* = 32)	30.63	20 (0, 95)	
**Lymph node metastasis**			0.141
Yes (*n* = 50)	47.12	70 (27.5, 120)	
No (*n* = 21)	33.33	10 (0, 105)	
**TNM stage**			0.957
Stage 1–2 (*n* = 12)	45.83	70 (0, 112.5)	
Stage 3 (*n* = 59)	42.47	60 (10,120)	

* *p < 0.05*.

Gastric cardia cancer tumors with higher MyD88 expression had higher histological grade (*p* = 0.041). There was no significant relationship between the expression of MyD88 and other clinical and pathological parameters in GCC.

All 70 patients followed were involved for survival analysis. On multivariable analysis, MyD88 did not correlate with survival in the GCC patients (overall survival, *p* = 0.828). Lymph node metastasis [hazard ratio (HR), 2.715; 95% confidence interval (CI), 1.348–5.468; *p* = 0.005] was independently associated with GCC patients' survival (Table 4).

**Table 4:** Multivariate analysis of factors associated with survival in GCC patients.

**Variable**	**HR (95% CI)**	***P*-value**
Lymph node metastasis (yes vs. no)	2.715 (1.348–5.469)	0.005^**^
Age		0.219
Sex (male/female)		0.172
Histology (well/moderate vs. poor)		0.071
MyD88		0.828
Length		0.856
TNM (III vs. I/II)		0.428

** *p < 0.01*.

## Discussion

The etiology of GCC is unclear. Previous reports showed that GCC is different from adenocarcinomas located in the lower esophagus or distal stomach in both epidemiology and biology (7). Gastric cardia cancer is defined as carcinoma in which the epicenter is ≤2 cm below the esophageal–gastric junction (32) in China. The highest regional rate of GCC was in Eastern/Southeastern Asia (1). The reason for a higher incidence of GCC in Chaoshan area in China is unknown. Given the differences between GCC and NGCC, in the present study, we considered GCC as a separate disease and reported for the first time in gastric cardia tissues the expression of MyD88 and its relationship with *H. pylori* infection and NF-κB p105/p50 expression. We observed that MyD88 expression gradually increased from normal tissue, gastric cardia inflammation, and carcinoma. A positive correlation between MyD88 and p105/p50 expression was detected. Thereby we provide pathological evidence that MyD88 expression is involved in gastric cardia tissue inflammation and carcinogenesis.

Lipopolysaccharide (LPS) is found in the outer membrane of *Helicobacter*, and it was reported that LPS could upregulate MyD88 expression. Few studies showed correlation between *H. pylori* and MyD88 expression in gastric cardia tissue. Here, we showed that MyD88 expression is higher in *H. pylori*–positive cases in comparison with *H. pylori*–negative cases, but the result was not significant. Several factors may contribute to lack of significant relationship between MyD88 expression and the *H. pylori* infection. One is the induction of endotoxin tolerance (33, 34). Lipopolysaccharide is the major component of *H. pylori*. Studies showed that after repeated challenge by LPS a reduced inflammatory response was observed, which is termed LPS tolerance. Lipopolysaccharide-induced tolerance can down-regulate the surface expression of the TLR4-MD2 complex, which might block MyD88-dependent pathways (35). We supposed that some of the patients with repeated *H. pylori* infection might reduce MyD88 expression. Second is that evidences suggest host genetics, environmental factors, and bacterial virulence factors might affect the ability of *H. pylori* to manipulate the immune response (33). These factors may contribute that some positive infection individuals show higher expression of MyD88 but not reach significant level. Third, pattern of MyD88 expression might not significantly change after the bacteria were eradicated. Michalkiewicz et al. (34) found that the involvement of *H. pylori* did not result in a significant upregulation of MyD88 mRNA expression when analyzing the expression of innate immunity components in the gastric mucosa among *H. pylori–*infected and uninfected children, which was consistent with our result.

Evidence showed that MyD88 can induce proinflammatory response and inflammation, which is regarded as the most important factor contributing to tumorigenesis (8, 21, 36). In the present study, MyD88 expression was evaluated from normal mucosa to inflammation and carcinoma restricted to gastric cardia tissue. MyD88 expression in normal cells differs in different tissue. Similar to the studies in gastric tissue (8, 37), our data indicated that the expression of MyD88 is low in normal gastric cardia tissue. MyD88 expression was increased during chronic inflammation. Echizen et al. (38) reported that depletion of MyD88 results in suppression of the inflammatory microenvironment in gastric tumors. These evidences indicated that MyD88 plays a role in increasing inflammation and changes innate immune activation between normal and mild inflammation. Gastric cardia cancer has the highest MyD88 expression and mainly in intestinal-type adenocarcinoma with inflammation. Gastric adenocarcinomas can be classified as the intestinal type and diffuse type according to the Lauren classification (39). Intestinal-type adenocarcinoma cells tend to form glands. Diffuse-type adenocarcinoma cells are poorly differentiated and tend to scatter throughout the stomach rather than form glands (40). Inflammatory cell infiltration was common in the intestinal-type adenocarcinoma in this study. We also showed that MyD88 expression was significantly higher in the well- and moderately differentiated tumors than in the poorly differentiated tumors, and most of the intestinal-type adenocarcinoma are well-differentiated. Studies reported that diffuse- and intestinal-type gastric carcinomas differ in risk factors, epidemiology, and distinct causal pathways (41–46). Our observation suggested that MyD88 pathway plays more important role in intestinal-type adenocarcinoma and might be responsible for the inflammatory response and carcinogenesis of this type of adenocarcinoma. The higher MyD88 expression in well-/moderately differentiated tumors comparing to poorly differentiated tumors might suggest that the MyD88 expression level is changing with the differentiation of cells. With the constant accumulation of different gene mutation and expression during tumor differentiation, MyD88 expression might be changing. We speculated that during tumor progression the antitumor role of MyD88 affects tumor differentiation to a certain degree resulting in well-/moderately differentiated tumors with higher MyD88 expression.

Reports have shown that MyD88 coupled with NF-κB contributes to carcinogenesis. Nuclear factor κB is the important signaling molecule downstream of MyD88 and data on how MyD88 deficiency affects carcinogenesis involved the role of NF-κB in cancer (21, 47). MyD88 is thought to mediate NF-κB activation and cytokine production (48, 49). Nuclear factor κB is able to regulate inflammation, cell differentiation, and apoptosis and plays a role in tumorigenesis (50–54). Nuclear factor κB p105/p50 usually locates in the cytoplasm. Adverse stimuli can activate NF-κB pathway, and p50 translocates into the nucleus then changes cell signaling (55). In the present study, we demonstrated positive expression of p105/p50 both in the cytoplasm and nucleus of GCC cells. We found that MyD88 had significantly positive relationship with NF-κB p105/p50, suggesting that p105/p50 and MyD88 are both involved in GCC tumorigenesis.

Different from the study results in hepatocellular carcinoma and epithelial ovarian cancer in which recurrence rate was higher and recurrence-free survival and overall survival were poorer in patients with MyD88 overexpression (8), we showed that the expression of MyD88 did not correlate with survival of GCC patients. The role of MyD88 in cancer prognosis might differ in different cancers. Lymph node metastasis was independently associated with GCC patient survival.

Although some previous studies proved that MyD88 has protective effects in gastric carcinogenesis (56). Our results provide evidences about the contribution of MyD88 in the regulation of inflammation, carcinogenesis, and tumor differentiation in gastric cardia tissue. Enhanced MyD88 expression was closely related with the intestinal-type carcinomas with inflammatory cell infiltration. Furthermore, NF-κB p105/p50 showed positive relationship with MyD88 expression in GCC tissue. The lack of significance of MyD88 as a prognostic factor in GCC might be due to the complex role of MyD88 in cancer tissue, and we need further studies to provide evidences.

## Data Availability Statement

The datasets used and analyzed during the current study are available from the corresponding author on reasonable request.

## Ethics Statement

The studies involving human participants were reviewed and approved by Ethical review committees of the Medical College of Shantou University. The patients/participants provided their written informed consent to participate in this study. Written informed consent was obtained from the individual(s) for the publication of any potentially identifiable images or data included in this article.

## Author Contributions

SL and GC designed the research study. JC, DX, WL, and DG conducted the experiments. GC and MX collected clinical data. SL, GC, and RS analyzed the data. SL wrote the manuscript with contribution from all authors. All authors read and approved the final version of the paper.

## Conflict of Interest

The authors declare that the research was conducted in the absence of any commercial or financial relationships that could be construed as a potential conflict of interest. The handling editor declared a shared affiliation, though no other collaboration, with the authors.
